# SPIRIT 2025 statement: Updated guideline for protocols of randomised trials

**DOI:** 10.1371/journal.pmed.1004589

**Published:** 2025-04-28

**Authors:** An-Wen Chan, Isabelle Boutron, Sally Hopewell, David Moher, Kenneth F. Schulz, Gary S. Collins, Ruth Tunn, Rakesh Aggarwal, Michael Berkwits, Jesse A. Berlin, Nita Bhandari, Nancy J. Butcher, Marion K. Campbell, Runcie C. W. Chidebe, Diana R. Elbourne, Andrew J. Farmer, Dean A. Fergusson, Robert M. Golub, Steven N. Goodman, Tammy C. Hoffmann, John P. A. Ioannidis, Brennan C. Kahan, Rachel L. Knowles, Sarah E. Lamb, Steff Lewis, Elizabeth Loder, Martin Offringa, Philippe Ravaud, Dawn P. Richards, Frank W. Rockhold, David L. Schriger, Nandi L. Siegfried, Sophie Staniszewska, Rod S. Taylor, Lehana Thabane, David J. Torgerson, Sunita Vohra, Ian R. White, Asbjørn Hróbjartsson

**Affiliations:** 1 Department of Medicine, Women’s College Research Institute, University of Toronto, Toronto, Ontario, Canada; 2 Université Paris Cité and Université Sorbonne Paris Nord, Inserm, INRAE, Centre for Research in Epidemiology and Statistics (CRESS), Paris, France; 3 Centre d’Epidémiologie Clinique, Hôpital Hôtel Dieu, AP-HP, Paris, France; 4 Oxford Clinical Trials Research Unit, Centre for Statistics in Medicine, University of Oxford, Oxford, United Kingdom; 5 Centre for Journalology, Clinical Epidemiology Programme, Ottawa Hospital Research Institute, Ottawa, Ontario, Canada; 6 Department of Obstetrics and Gynecology, School of Medicine, University of North Carolina at Chapel Hill, Chapel Hill, North Carolina, United States of America; 7 UK EQUATOR Centre, Centre for Statistics in Medicine, University of Oxford, Oxford, United Kingdom; 8 Jawaharlal Institute of Postgraduate Medical Education and Research, Puducherry, India; 9 Office of Science Dissemination, Centers for Disease Control and Prevention, Atlanta, Georgia, United States of America; 10 Department of Biostatistics and Epidemiology, School of Public Health, Center for Pharmacoepidemiology and Treatment Science, Rutgers University, New Brunswick, New Jersey, United States of America; 11 JAMA Network Open, Chicago, Illinois, United States of America; 12 Centre for Health Research and Development, Society for Applied Studies, New Delhi, India; 13 Child Health Evaluation Services, The Hospital for Sick Children Research Institute, Toronto, Ontario, Canada; 14 Department of Psychiatry, University of Toronto, Toronto, Ontario, Canada; 15 Aberdeen Centre for Evaluation, University of Aberdeen, Aberdeen, United Kingdom; 16 Project PINK BLUE—Health & Psychological Trust Centre, Utako, Abuja, Nigeria; 17 Department of Sociology and Gerontology, Miami University, Ohio, United States of America; 18 Department of Medical Statistics, London School of Hygiene and Tropical Medicine, London, United Kingdom; 19 Nuffield Department of Primary Care Health Sciences, University of Oxford, Oxford, United Kingdom; 20 Ottawa Hospital Research Institute, Ottawa, Ontario, Canada; 21 Department of Medicine, Northwestern University Feinberg School of Medicine, Chicago, Illinois, United States of America; 22 Department of Epidemiology and Population Health, Stanford University, Palo Alto, California, United States of America; 23 Institute for Evidence-Based Healthcare, Faculty of Health Sciences and Medicine, Bond University, University Drive, Robina, Queensland, Australia; 24 Departments of Medicine, of Epidemiology and Population Health, of Biomedical Data Science, and of Statistics, and Meta-Research Innovation Center at Stanford (METRICS), Stanford University, Stanford, California, United States of America; 25 MRC Clinical Trials Unit at University College London, London, United Kingdom; 26 University College London, UCL Great Ormond Street Institute of Child Health, London, United Kingdom; 27 NIHR Exeter Biomedical Research Centre, Faculty of Health and Life Sciences, University of Exeter, Exeter, United Kingdom; 28 Edinburgh Clinical Trials Unit, Usher Institute-University of Edinburgh, Edinburgh BioQuarter, Edinburgh, United Kingdom; 29 The BMJ, BMA House, London, United Kingdom; 30 Harvard Medical School, Boston, Massachusetts, United States of America; 31 Université Paris Cité, Inserm, INRAE, Centre de Recherche Epidémiologie et Statistiques, Université Paris Cité, Paris, France; 32 Clinical Trials Ontario, MaRS Centre, Toronto, Ontario, Canada; 33 Duke Clinical Research Institute, Duke University Medical Center, Durham, North Carolina, United States of America; 34 Department of Emergency Medicine, University of California, Los Angeles, California, United States of America; 35 South African Medical Research Council, Cape Town, South Africa; 36 Warwick Applied Health, Warwick Medical School, University of Warwick, Coventry, United Kingdom; 37 MRC/CSO Social and Public Health Sciences Unit & Robertson Centre for Biostatistics, Institute of Health and Wellbeing, University of Glasgow, Glasgow, United Kingdom; 38 Department of Health Research Methods Evidence and Impact, McMaster University, Hamilton, Ontario, Canada; 39 St Joseph’s Healthcare Hamilton, Hamilton, Ontario, Canada; 40 York Trials Unit, Department of Health Sciences, University of York, York, United Kingdom; 41 Faculty of Medicine and Dentistry, University of Alberta, Edmonton, Alberta, Canada; 42 Centre for Evidence-Based Medicine Odense and Cochrane Denmark, Department of Clinical Research, University of Southern Denmark, Odense, Denmark; 43 Open Patient data Explorative Network, Odense University Hospital, Odense, Denmark; Heidelberg University, GERMANY

## Abstract

**Importance:**

The protocol of a randomised trial is the foundation for study planning, conduct, reporting, and external review. However, trial protocols vary in their completeness and often do not address key elements of design and conduct. The SPIRIT (Standard Protocol Items: Recommendations for Interventional Trials) statement was first published in 2013 as guidance to improve the completeness of trial protocols. Periodic updates incorporating the latest evidence and best practices are needed to ensure that the guidance remains relevant to users.

**Objective:**

To systematically update the SPIRIT recommendations for minimum items to address in the protocol of a randomised trial.

**Design:**

We completed a scoping review and developed a project specific database of empirical and theoretical evidence to generate a list of potential changes to the SPIRIT 2013 checklist. The list was enriched with recommendations provided by lead authors of existing SPIRIT/CONSORT (Consolidated Standards of Reporting Trials) extensions (Harms, Outcomes, Non-pharmacological Treatment) and other reporting guidelines (TIDieR). The potential modifications were rated in a three-round Delphi survey followed by a consensus meeting.

**Findings:**

Overall, 317 individuals participated in the Delphi consensus process and 30 experts attended the consensus meeting. The process led to the addition of two new protocol items, revision to five items, deletion/merger of five items, and integration of key items from other relevant reporting guidelines. Notable changes include a new open science section, additional emphasis on the assessment of harms and description of interventions and comparators, and a new item on how patients and the public will be involved in trial design, conduct, and reporting. The updated SPIRIT 2025 statement consists of an evidence-based checklist of 34 minimum items to address in a trial protocol, along with a diagram illustrating the schedule of enrolment, interventions, and assessments for trial participants. To facilitate implementation, we also developed an expanded version of the SPIRIT 2025 checklist and an accompanying explanation and elaboration document.

**Conclusions and relevance:**

Widespread endorsement and adherence to the updated SPIRIT 2025 statement have the potential to enhance the transparency and completeness of trial protocols for the benefit of investigators, trial participants, patients, funders, research ethics committees, journals, trial registries, policymakers, regulators, and other reviewers.

## Summary

- A complete, transparent, and accessible protocol is critical for the planning, conduct, reporting, and external review of randomised trials
- The SPIRIT (Standard Protocol Items: Recommendations for Interventional Trials) 2025 statement provides guidance on items to address in trial protocols, reflecting methodological advances and feedback from users
- The SPIRIT 2025 statement consists of a checklist of 34 minimum items; a diagram illustrating the schedule of enrolment, interventions, and assessments; and an expanded checklist that details the critical elements of each checklist item
- Research teams, sponsors, editors, funders, and research ethics committees/institutional review boards should use and endorse SPIRIT 2025 to promote transparent protocol content

## Introduction

“Readers should not have to infer what was probably done; they should be told explicitly.” Douglas G Altman [1]

Robustly designed, properly conducted, and fully reported randomised trials underpin evidence-based practice and policy. As the most important record of planned methods and conduct, a well written protocol has a key role in promoting consistent and rigorous execution by the trial team. The protocol also serves as the basis for oversight and review of scientific, ethical, safety, and operational issues by funders, regulators, research ethics committees/institutional review boards (REC/IRB), journal editors, researchers, patients, and the public [2–9]. After trial completion, the protocol is essential for understanding and interpreting the results.

Despite the central role of protocols, there is substantial variation in the completeness of protocol content [10,11]. Many trial protocols do not adequately describe important elements including the primary outcomes, treatment allocation methods, use of blinding, measurement of adverse events, sample size calculations, data analysis methods, dissemination policies, and roles of sponsors and investigators in trial design [10–12]. Gaps in protocol content can lead to avoidable protocol amendments [13], inconsistent or poor trial conduct, and lack of transparency in terms of what was planned and implemented.

In response to these protocol deficiencies, the SPIRIT (Standard Protocol Items: Recommendations for Interventional Trials) guidance was first published in 2013 [14,15]. Aligned with the CONSORT (Consolidated Standards of Reporting Trials) guidance for reporting completed trials [16], the international SPIRIT initiative aims to improve the completeness of trial protocols by producing evidence-based recommendations for a minimum set of items to be addressed in protocols. The SPIRIT 2013 guidance has been translated into seven languages and is widely endorsed by national funders, research organisations, over 150 medical journals, and the World Association of Medical Editors.

In January 2020, the SPIRIT and CONSORT executive groups held a joint meeting in Oxford, UK, to discuss strategic planning. There was broad recognition of the need to update both checklists to reflect the evolving trials environment and methodological advancements, including the growing international support for improved research transparency, accessibility, and reproducibility (collectively referred to as open science) [17] as well as greater patient and public involvement in research.

As the SPIRIT 2013 and CONSORT 2010 statements were conceptually linked with overlapping content and implementation strategies, the two groups decided to merge into the joint SPIRIT-CONSORT executive group and to update both checklists simultaneously. The joint update was an opportunity to further align the checklists and provide consistent guidance in the reporting of trial design, conduct, and analysis—from study conception to the publication of results. Harmonising the reporting recommendations could help improve usability and adherence [18]. Here, we introduce the updated SPIRIT 2025 statement; the CONSORT 2025 statement is published separately [16].

## Methods

The methods have been detailed elsewhere [19,20]. In brief, we followed the EQUATOR Network guidance for developers of health research guidelines [21]. We first conducted a scoping review of the literature from 2013 to 2022 to identify published comments suggesting modifications or reflecting on the strengths and challenges of SPIRIT 2013; these findings have been published separately [22]. We also conducted a broader search for empirical and theoretical evidence published from 2013 to 2024 that was relevant to SPIRIT and risk of bias in randomised trials, producing the SPIRIT-CONSORT Evidence Bibliographic database [23]. The evidence identified in the literature was combined with recommendations provided by the lead authors of key SPIRIT and CONSORT extensions (Harms [24], Outcomes [25], Non-pharmacological Treatment [26]), and the Template for Intervention Description and Replication (TIDieR) [27], along with user feedback.

Based on the gathered evidence, a preliminary list of five potential additions to the SPIRIT 2013 checklist was created for review in an international, three-round online Delphi survey. A total of 317 participants were recruited through professional research networks, societies, and the project website. Participants represented a broad range of roles in clinical trials, including statisticians/methodologists/epidemiologists (n = 198), trial investigators (n = 73), systematic reviewers/guideline developers (n = 73), clinicians (n = 58), journal editors (n = 47), and patients and members of the public (n = 17) (numbers are not mutually exclusive). During each survey round, participants rated the importance of modifications on a five-point Likert scale and provided comments or suggestions for additional items. A high level of agreement was defined by at least 80% of respondents rating the importance of a proposed modification as high (score of 4 or 5) or low (score of 1 or 2).

The Delphi survey results were then discussed at a two-day online consensus meeting in March 2023, attended by 30 invited international experts representing a range of relevant groups. Meeting participants discussed potential new and modified SPIRIT checklist items, with anonymous polling of participants in cases of ongoing disagreement.

The executive group met in person in April 2023 to develop a draft checklist based on the consensus meeting discussion. After a further round of review by consensus meeting participants, the executive group finalised the SPIRIT 2025 statement.

### Updated SPIRIT 2025 statement

**Fig 1:**
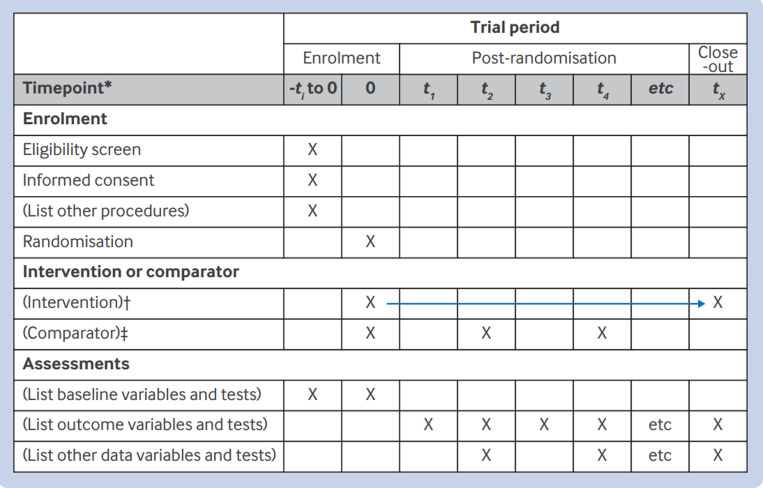
SPIRIT 2025 diagram of the schedule of enrolment, interventions, and assessments. Recommended content can be displayed using various schematic formats. *List target time points and acceptable time windows in this row (e.g., 30 ± 3 days). †Arrow indicates continuous delivery of intervention (e.g., drug). ‡Example illustrates delivery of comparator at discrete time points (e.g., psychotherapy).

The SPIRIT 2025 statement comprises a checklist of 34 minimum protocol items (Table 1) and a diagram illustrating the schedule of enrolment, interventions, and assessments (Fig 1). An accompanying SPIRIT 2025 explanation and elaboration document provides background and context for each checklist item along with examples of good reporting [28]. We strongly recommend that the SPIRIT 2025 explanation and elaboration document be used routinely alongside the SPIRIT 2025 statement to facilitate better understanding of and adherence to the checklist items.

**Table 1:** SPIRIT 2025 checklist of items to address in a randomised trial protocol.

Section/topic	No	SPIRIT 2025 checklist item description
**Administrative information**		
Title and structured summary	1a	Title stating the trial design, population, and interventions, with identification as a protocol
	1b	Structured summary of trial design and methods, including items from the World Health Organization Trial Registration Data Set
Protocol version	2	Version date and identifier
Roles and responsibilities	3a	Names, affiliations, and roles of protocol contributors
	3b	Name and contact information for the trial sponsor
	3c	Role of trial sponsor and funders in design, conduct, analysis, and reporting of trial; including any authority over these activities
	3d	Composition, roles, and responsibilities of the coordinating site, steering committee, endpoint adjudication committee, data management team, and other individuals or groups overseeing the trial, if applicable
**Open science**		
Trial registration	4	Name of trial registry, identifying number (with URL), and date of registration. If not yet registered, name of intended registry
Protocol and statistical analysis plan	5	Where the trial protocol and statistical analysis plan can be accessed
Data sharing	6	Where and how the individual de-identified participant data (including data dictionary), statistical code, and any other materials will be accessible
Funding and conflicts of interest	7a	Sources of funding and other support (e.g., supply of drugs)
	7b	Financial and other conflicts of interest for principal investigators and steering committee members
Dissemination policy	8	Plans to communicate trial results to participants, healthcare professionals, the public, and other relevant groups (e.g., reporting in trial registry, plain language summary, publication)
**Introduction**		
Background and rationale	9a	Scientific background and rationale, including summary of relevant studies (published and unpublished) examining benefits and harms for each intervention
	9b	Explanation for choice of comparator
Objectives	10	Specific objectives related to benefits and harms
**Methods: Patient and public involvement, trial design**		
Patient and public involvement	11	Details of, or plans for, patient or public involvement in the design, conduct, and reporting of the trial
Trial design	12	Description of trial design including type of trial (e.g., parallel group, crossover), allocation ratio, and framework (e.g., superiority, equivalence, non-inferiority, exploratory)
**Methods: Participants, interventions, and outcomes**		
Trial setting	13	Settings (e.g., community, hospital) and locations (e.g., countries, sites) where the trial will be conducted
Eligibility criteria	14a	Eligibility criteria for participants
	14b	If applicable, eligibility criteria for sites and for individuals who will deliver the interventions (e.g., surgeons, physiotherapists)
Intervention and comparator	15a	Intervention and comparator with sufficient details to allow replication including how, when, and by whom they will be administered. If relevant, where additional materials describing the intervention and comparator (e.g., intervention manual) can be accessed
	15b	Criteria for discontinuing or modifying allocated intervention/comparator for a trial participant (e.g., drug dose change in response to harms, participant request, or improving/worsening disease)
	15c	Strategies to improve adherence to intervention/comparator protocols, if applicable, and any procedures for monitoring adherence (e.g., drug tablet return, sessions attended)
	15d	Concomitant care that is permitted or prohibited during the trial
Outcomes	16	Primary and secondary outcomes, including the specific measurement variable (e.g., systolic blood pressure), analysis metric (e.g., change from baseline, final value, time to event), method of aggregation (e.g., median, proportion), and time point for each outcome
Harms	17	How harms are defined and will be assessed (e.g., systematically, non-systematically)
Participant timeline	18	Time schedule of enrolment, interventions (including any run-ins and washouts), assessments, and visits for participants. A schematic diagram is highly recommended (see Fig 1)
Sample size	19	How sample size was determined, including all assumptions supporting the sample size calculation
Recruitment	20	Strategies for achieving adequate participant enrolment to reach target sample size
**Methods: Assignment of interventions**		
Randomisation:		
Sequence generation	21a	Who will generate the random allocation sequence and the method used
	21b	Type of randomisation (simple or restricted) and details of any factors for stratification. To reduce predictability of a random sequence, other details of any planned restriction (e.g., blocking) should be provided in a separate document that is unavailable to those who enrol participants or assign interventions
Allocation concealment mechanism	22	Mechanism used to implement the random allocation sequence (e.g., central computer/telephone; sequentially numbered, opaque, sealed containers), describing any steps to conceal the sequence until interventions are assigned
Implementation	23	Whether the personnel who will enrol and those who will assign participants to the interventions will have access to the random allocation sequence
Blinding	24a	Who will be blinded after assignment to interventions (e.g., participants, care providers, outcome assessors, data analysts)
	24b	If blinded, how blinding will be achieved and description of the similarity of interventions
	24c	If blinded, circumstances under which unblinding is permissible, and procedure for revealing a participant’s allocated intervention during the trial
**Methods: Data collection, management, and analysis**		
Data collection methods	25a	Plans for assessment and collection of trial data, including any related processes to promote data quality (e.g., duplicate measurements, training of assessors) and a description of trial instruments (e.g., questionnaires, laboratory tests) along with their reliability and validity, if known. Reference to where data collection forms can be accessed, if not in the protocol
	25b	Plans to promote participant retention and complete follow-up, including list of any outcome data to be collected for participants who discontinue or deviate from intervention protocols
Data management	26	Plans for data entry, coding, security, and storage, including any related processes to promote data quality (e.g., double data entry; range checks for data values). Reference to where details of data management procedures can be accessed, if not in the protocol
Statistical methods	27a	Statistical methods used to compare groups for primary and secondary outcomes, including harms
	27b	Definition of who will be included in each analysis (e.g., all randomised participants), and in which group
	27c	How missing data will be handled in the analysis
	27d	Methods for any additional analyses (e.g., subgroup and sensitivity analyses)
**Methods: Monitoring**		
Data monitoring committee	28a	Composition of data monitoring committee (DMC); summary of its role and reporting structure; statement of whether it is independent from the sponsor and funder; conflicts of interest and reference to where further details about its charter can be found, if not in the protocol. Alternatively, an explanation of why a DMC is not needed
	28b	Explanation of any interim analyses and stopping guidelines, including who will have access to these interim results and make the final decision to terminate the trial
Trial monitoring	29	Frequency and procedures for monitoring trial conduct. If there is no monitoring, give explanation
**Ethics**		
Research ethics approval	30	Plans for seeking research ethics committee/institutional review board approval
Protocol amendments	31	Plans for communicating important protocol modifications to relevant parties
Consent or assent	32a	Who will obtain informed consent or assent from potential trial participants or authorised proxies, and how
	32b	Additional consent provisions for collection and use of participant data and biological specimens in ancillary studies, if applicable
Confidentiality	33	How personal information about potential and enrolled participants will be collected, shared, and maintained in order to protect confidentiality before, during, and after the trial
Ancillary and post-trial care	34	Provisions, if any, for ancillary and post-trial care, and for compensation to those who suffer harm from trial participation

SPIRIT = Standard Protocol Items: Recommendations for Interventional Trials.

To present the recommendations in diverse formats, we also developed an expanded version of the SPIRIT 2025 checklist with bullet points of key issues to consider for each item (S1 Appendix), as done with other initiatives [29–31]. The expanded checklist comprises an abridged version of elements presented in the SPIRIT 2025 explanation and elaboration document [28], with examples and references removed.

### Main changes

Substantive changes made in this update are detailed in Box 1. We added two new checklist items, revised the content of five items, deleted three items, merged two items, and integrated key items from CONSORT Harms 2022 [24], SPIRIT-Outcomes 2022 [25], and TIDieR [27] into the main checklist and explanatory document. We also restructured the SPIRIT checklist and created a new open science section consolidating items critical to promoting access to information about trial methods and results, including trial registration; sharing of the full protocol, statistical analysis plan, and de-identified participant level data; and disclosure of funding sources and conflicts of interest. We have also harmonised the wording between SPIRIT and CONSORT checklist items and clarified the wording of some items. A comparison of the SPIRIT 2025 and 2013 checklists is available in S2 Appendix.

**Box 1: Summary of main changes in SPIRIT 2025**

**Addition of new checklist items:**

- Item 11: added item on how patients and the public are involved in the design, conduct, and reporting of the trial
- Item 29: added item on trial monitoring (replaces prior item on auditing)

**Revised content of checklist items**

- Item 4: revised item to include date of trial registration
- Item 5: revised item to include where the statistical analysis plan can be accessed in addition to the trial protocol (previously covered under item on statistical methods)
- Item 7b: revised item to include financial and other conflicts of interest of steering committee members
- Item 24a/24b: split item into separate sub-items covering (a) who will be blinded and (b) how, and revised to include description of the similarity of interventions
- Item 27d: revised to refer to sensitivity analyses

**Deletion/merger of checklist items**

- Deleted item on auditing (replaced with trial monitoring)
- Deleted appendix items:
  - Informed consent materials
  - Biological specimens
- Merged item on access to data for trial investigators with item on data management (item 26)
- Merged item on authorship eligibility guidelines and use of professional writers with item on dissemination policy (item 8)

**Integration of checklist items from CONSORT Harms 2022 and TIDieR**

- Revised items to emphasise reporting of harms (items 10, 17, 27a) [24] and to call for additional details relating to interventions and comparators (item 15a) [27]

**Structure and organisation of checklist items**

- Created a new section on open science that includes trial registration (item 4), access to the trial protocol and statistical analysis plan (item 5), plans for sharing de-identified participant level data (item 6), funding and conflicts of interest (item 7), and plans for dissemination of trial results (item 8)
- Item 14a/b: split item into separate sub-items covering eligibility criteria for (a) participants and (b) sites and personnel
- Item 27b/27c: split item into separate sub-items covering the analysis population and methods for handling missing data
- Aligned wording of SPIRIT checklist items with that of CONSORT checklist items, and vice versa
- Clarified and simplified wording of some items.

CONSORT = Consolidated Standards of Reporting Trials; SPIRIT = Standard Protocol Items: Recommendations for Interventional Trials; TIDieR = Template for Intervention Description and Replication.

### Definition of a randomised trial protocol

The protocol is a central document that provides sufficient detail to enable (a) understanding of the rationale, objectives, population, interventions, methods, statistical analyses, ethical considerations, dissemination plans, and administration of the trial; (b) replication of trial methods and conduct; and (c) appraisal of trial validity, feasibility, and ethical rigour [14].

The full protocol must be submitted for approval by an REC/IRB before enrolling participants [32]. As a living document that is often formally amended during the trial [13,33], every protocol version should contain a transparent audit trail documenting the dates and descriptions of changes. Important protocol amendments should be reported to REC/IRBs and trial registries as they occur, and subsequently described in reports of completed trials [34].

### Scope of SPIRIT 2025

SPIRIT 2025 addresses the minimum content of a protocol, focusing on the most common type of randomised trial—the two-group parallel design. However, most of the SPIRIT items are relevant to any type of trial. SPIRIT 2025 has been designed to complement and enhance the expanding trial registration requirements mandated by legislation, journals, and funding policies [35]. SPIRIT 2025 encompasses and builds upon recommendations from the International Council for Harmonization Good Clinical Practice E6(R3) guidance [36] and 2024 Declaration of Helsinki [32], including the Declaration of Helsinki’s requirement that the protocol address potential conflicts of interest and provision of post-trial care.

It is feasible to address all SPIRIT 2025 checklist items in a single protocol document, as illustrated by the examples we identified from existing protocols for every item [28]. There are often related documents (e.g., full statistical analysis plan [37], data management plan) that provide further details on specific items. Any such documents should be referenced in the protocol and made available for review.

The main purpose of SPIRIT 2025 is to promote transparency and an adequate description of what is planned—not to prescribe how a trial should be designed or conducted. The checklist also does not focus on the protocol format, which is often subject to local regulations or practice. The checklist should not be used to appraise the quality of trial design or conduct, as it is possible for the protocol of a poorly designed trial to address all checklist items by fully describing its inadequate design and conduct features. Recent guidance from the World Health Organization (WHO) outlines best practices for designing and conducting trials [38].

### Implementation

The SPIRIT 2025 statement supersedes the SPIRIT 2013 statement, which should no longer be used or cited. We encourage research organisations, sponsors, funders, REC/IRBs, journal editors and publishers to endorse SPIRIT 2025 and request that they update their resources and instructions to research teams and reviewers with reference to the updated guidance.

When protocols are submitted for review or publication, we recommend the submission of a completed SPIRIT 2025 checklist that indicates where (e.g., page number) checklist items are reported in the protocol. Trial investigators and sponsors should address all SPIRIT 2025 checklist items in the protocol before REC/IRB submission. If an item is not relevant for a particular trial (e.g., no interim analysis planned), then this should be explicitly stated along with an explanation. We encourage investigators to ensure consistency of information in the protocol, related documents (e.g., full statistical analysis plan) [37], and trial registry record [39].

To facilitate implementation, a new SPIRIT-CONSORT website (consort-spirit.org) provides resources based on the SPIRIT and CONSORT 2025 statements, including a fillable checklist, protocol writing tools, and training materials for researchers, trainees, journal editors, peer reviewers, patients, and the public.

## Limitations

As a minimum standard focused on parallel group randomised trials, SPIRIT 2025 may not encompass every protocol item relevant for a particular trial. For example, a factorial trial design has additional analytical considerations related to potential statistical interactions [40], and trials evaluating patient reported outcomes have specific considerations regarding data collection methods [41]. Extensions to SPIRIT 2013 were developed to provide additional guidance on reporting different types of trial designs, data, and interventions [25,34,40–47]. We will engage with the leaders of these extensions to implement a process for aligning them with the updated SPIRIT 2025 statement. In the meantime, we recommend that the existing version of the relevant SPIRIT extensions be used.

### Potential impact

The updated SPIRIT 2025 statement and its accompanying explanation and elaboration document can be helpful in several ways. SPIRIT 2025 will continue to serve as an educational resource for new investigators, trainees, peer reviewers, and REC/IRB members. The explicit incorporation of an open science section in the SPIRIT checklist will support the growing global push for greater transparency and sharing of trial materials and outputs to facilitate evidence synthesis and reproducibility of research.

Trial investigators can consult the guidance when drafting their protocols to ensure that all elements are addressed. Meta-research reviews of protocols have found improved completeness of protocol content after SPIRIT 2013 was introduced [10,11,48,49]. In addition to improved reporting, adherence to SPIRIT 2025 may promote high quality trial design and implementation because SPIRIT is used during the planning stage of a trial. This provides an opportunity to improve the validity and successful completion of trials by reminding investigators about important issues to consider before the study begins. Better protocols can also help study personnel to implement the trial consistently across sites.

Another potential benefit of SPIRIT 2025 is its impact on administrative burden. Improved completeness of protocols may improve the efficiency of external review by reducing avoidable queries to investigators about incomplete or unclear protocol related information [50,51]. High quality protocols addressing all SPIRIT items may also help to reduce the number and burden of protocol amendments during the trial—many of which can be avoided with careful consideration of key issues when developing the protocol [13,33]. Widespread adoption of SPIRIT 2025 as a common standard across REC/IRBs, funding agencies, regulatory agencies, and journals could simplify the work of trial investigators and sponsors because a SPIRIT based protocol would then fulfil the harmonised application requirements of multiple groups.

Further, adherence to SPIRIT 2025 may help ensure that protocols contain the requisite information for critical appraisal and trial interpretation by peer reviewers, funders, REC/IRBs, and journals [7]. High quality protocols provide important information about trial methods and conduct that is usually not available in trial registries or publications reporting completed trials. As a transparent record of the investigators’ original intent, comparison of protocols with reports of completed trials helps to identify selective reporting of results and undisclosed amendments, such as changes to primary outcomes or analyses [52,53]. These benefits of SPIRIT based protocols can only be fully realised when trial protocols are routinely made publicly available through trial registries (e.g., PDF upload), journals, and online repositories [7,54,55].

The SPIRIT 2025 statement incorporates new evidence and emerging perspectives to ensure that the guidance remains relevant to users. Widespread endorsement and adoption of the updated recommendations have the potential to improve protocol content and implementation; facilitate registration, oversight, and appraisal of trials; and ultimately enhance transparency and translation to better healthcare.

## Supporting information

S1 Appendix SPIRIT 2025 expanded checklist detailing items to address in randomised trial protocols, based on Explanation and Elaboration document. (DOCX)

S2 Appendix Comparison of SPIRIT 2025 and SPIRIT 2013 checklists. (DOCX)
